# Predicting evolution in response to climate change: the example of sprouting probability in three dormancy-prone orchid species

**DOI:** 10.1098/rsos.160647

**Published:** 2017-01-18

**Authors:** Richard P. Shefferson, Ryo Mizuta, Michael J. Hutchings

**Affiliations:** 1 Organization for Programs on Environmental Sciences, University of Tokyo, Meguro-ku, Tokyo, Japan; 2 Meteorological Research Institute, Tsukuba, Japan; 3 School of Life Sciences, University of Sussex, Falmer, Brighton, Sussex BN1 9QGUK

**Keywords:** adaptive dynamics, climate change, demography, evolutionarily stable strategy, game theory, Orchidaceae

## Abstract

Although many ecological properties of species respond to climate change, their evolutionary responses are poorly understood. Here, we use data from long-term demographic studies to predict evolutionary responses of three herbaceous perennial orchid species, *Cypripedium parviflorum*, *C. candidum* and *Ophrys sphegodes*, to predicted climate changes in the habitats they occupy. We focus on the evolution of sprouting probability, because all three species exhibit long-term vegetative dormancy, i.e. individual plants may not emerge above-ground, potentially for several consecutive years. The drivers of all major vital rates for populations of the species were analysed with general linear mixed models (GLMMs). High-dimensionality function-based matrix projection models were then developed to serve as core elements of deterministic and stochastic adaptive dynamics models used to analyse the adaptive context of sprouting in all populations. We then used regional climate forecasts, derived from high-resolution general atmospheric circulation models, of increased mean annual temperatures and spring precipitation at the occupied sites, to predict evolutionary trends in sprouting. The models predicted that *C. parviflorum* and *O. sphegodes* will evolve higher and lower probabilities of sprouting, respectively, by the end of the twenty-first century, whereas, after considerable variation, the probability of sprouting in *C. candidum* will return to its current level. These trends appear to be driven by relationships between mortality and size: in *C. parviflorum* and *C. candidum*, mortality is negatively related to size in the current year but positively related to growth since the previous year, whereas in *O. sphegodes*, mortality is positively related to size.

## 1. Introduction

The ecological and evolutionary consequences of climate change are of considerable concern for the conservation of species, populations and communities. In recent times, rapidly rising mean temperatures have altered the distributions of populations and communities, typically causing shifts to higher latitudes and altitudes [1] and changing flowering times, migration times and developmental schedules [2–4]. Many of these changes are detrimental [5]. For example, plant–pollinator and predator–prey interactions have been disrupted by alterations in species' phenologies [6–9]. However, analysis of the impacts of contemporary climate change is only now starting to move from documentation to explanation of its effects [10,11].

Evans *et al*. [12] and Mouquet *et al*. [13] have called for studies involving predictive modelling of the effects of climate change on species, populations and communities. Although climatologists have made considerable progress in developing global models to predict environmental disasters [14,15], ecologists have been slower to adopt a similar approach (but see [16]), though some models have been developed to predict species distribution changes [17], extinction risks [10] and alterations to biogeochemical cycles and ecosystem functioning [18]. Predictive models in ecology rarely feature the possibility of Darwinian evolution, originally because evolution was assumed to be irrelevant in short- to medium-term predictive models [12], but more recently because it is considered difficult to incorporate the complexity of evolution into models of ecological processes, particularly because of the need for large amounts of relevant data with which to parametrize models of evolutionary change. It is now recognized, however, that significant evolutionary and ecological changes can occur over similar time scales [19,20], suggesting that evolution could respond rapidly to climate change [21,22], and two-way interactions between evolution and ecological processes are probably common [20,23]. Predictions of alterations in species distributions, flowering times and species traits may therefore be inaccurate unless evolution in traits that can undergo strong selection in response to climate change is taken into consideration.

Ultimately, the fates of individual organisms scale up to influence many of the processes of interest when predicting species' responses to climate change. Life-history traits are likely to exert significant influences on population dynamics, because of the strong, direct relationships between fitness and demography [24], and these influences may depend on changes in mean climatic variables, and on variation about mean values [22]. Predicting life-history evolution therefore requires the integration of validated evolutionary models of the expected changes in traits of interest with accurate, predictive climatic models. The output of such models will aid the development of management strategies for populations of conservation concern.

Among the least understood life-history stages in herbaceous plants is vegetative dormancy. Instead of sprouting every year, individual plants of species that exhibit this phenomenon may remain underground in a rhizomatous or tuberous state, potentially for several consecutive years, before re-emerging [25,26]. For many years, this condition was assumed to be either maladaptive, or adaptive as a bet-hedging trait [27]. However, more recent data suggest that vegetative dormancy can be either an adaptive response to environmental stress or life-history costs associated with sprouting, as in *Cypripedium parviflorum* [28,29], or a bet-hedging trait in response to unpredictable risks in the above-ground environment, as in *Astragalus scaphoides* [30,31]. Consequently, the deterministic, directional nature of climate change may present a gradual increase in stress for dormancy-prone species, resulting in selection for increased vegetative dormancy over time.

In this paper, we analyse life-history evolution in response to climate change in three rare plant species, *C. parviflorum*, *C. candidum* and *Ophrys sphegodes* (all Orchidaceae). We focus on the evolution of sprouting probability because: (i) all of these species are capable of vegetative dormancy and (ii) vegetative dormancy, its causes and its evolutionary benefits are still poorly understood. The results of this study may, therefore, provide insights into its evolution and ecological function [26]. Shefferson *et al*. [28] developed adaptive dynamics models, based on life histories of individual plants across 3-year periods, that accurately predict observed sprouting probabilities in *C. parviflorum*. Unlike matrix and integral projection models, which are commonly used to parametrize adaptive dynamics models [22,32], these models included costs of growth and long-term costs of reproduction [28]. We now extend these models to include observed sprouting responses to annual variation in climatic variables. We then use climate predictions from two state-of-the-art, spatially high-resolution general atmospheric circulation models [33,34], to predict patterns in the evolution of sprouting probabilities under realistic climate change scenarios. Because vegetative dormancy is believed to be a response to stress [29], we hypothesize that sprouting probability will decline as a buffer against the risk of greater mortality in the face of the changing, novel climate conditions predicted for the future.

## 2. Material and methods

### 2.1. Study species and sites

We studied one population each of *C. parviflorum* Salisb., *C. candidum* Muhl. ex. Willd., and *O. sphegodes* Mill (hereafter, *C. parviflorum*, *C. candidum* and *Ophrys*, respectively). These species begin life as dust seeds, and germinate in association with an appropriate mycorrhizal fungus [35]. Germination leads to a life stage termed a protocorm, in which the plant is non-photosynthetic, mycotrophic and subterranean [35]. In *Cypripedium* species, the protocorm stage may last for 3 or more years [36]. In *Ophrys*, the protocorm is thought to last for up to 2 years before developing into a seedling [37]. Seedlings of all species may sprout or remain completely subterranean in any year. *Cypripedium* spp. are capable of living as a juvenile for up to 10 years before developing into adult plants, although the exact number of years varies between individuals (electronic supplementary material, figure S1*a*). Vegetative dormancy of up to 15 years has been documented in adult plants [28]. In *Ophrys*, most episodes of dormancy are less than or equal to 2 years, although dormancy of up to 8 years has been recorded (electronic supplementary material, figure S1*b*) [38].

Sympatric populations of *Cypripedium candidum* and *C*. *parviflorum* were monitored every year from 1994 to 2014 in a wet alkaline meadow at Gavin Prairie Nature Preserve, northeastern Illinois, USA. One *Ophrys* population was monitored every year from 1975 to 2006 at Castle Hill National Nature Reserve, Sussex, England. Gavin Prairie is characterized by communities dominated by tallgrass and *Quercus* spp. in drier areas, and by *Carex* spp. with some *Salix* spp. in wetter, tussock-filled areas. The vegetation at Castle Hill is ancient sheep-grazed, species-rich, calcareous grassland. Further site details are provided by Shefferson *et al*. [39,40] and Hutchings [38].

### 2.2. Field methods

*Cypripedium* populations were monitored every year at the start of flowering, in mid-May to early-June. We mapped the positions of all emergent plants, and then searched for others that had been observed in previous years. We define a sprout in *Cypripedium* as a single stem originating from a bud at the end of a rhizome. An individual plant can have several sprouts if the rhizome has branched, or if two buds form at rhizome tips. The number of sprouts produced by every plant, and the number of flowers on each sprout, was recorded. We could not search for seedlings, because they are both difficult to find in the tallgrass vegetation, and because the timing of their sprouting is unpredictable. In July of every year from 2000 onwards, we also recorded the number of fruits produced by each sprout.

*Ophrys* was recorded in a permanently marked 20 × 20 m quadrat. The position of every emergent plant was recorded in each year of the study by triangulation from two of the quadrat corners. Individual plants produce a single rosette of leaves, which is termed a sprout in this study. We recorded whether each plant was vegetative or flowering, the numbers of leaves and, for flowering plants, the number of flowers produced.

Life histories of all plants recorded during the field studies, including years spent in vegetative dormancy, were reconstructed for all three species. For further details of field methods, including criteria for determining whether plants were dormant or dead, see Shefferson *et al*. [27,39] and Hutchings [38,41,42].

### 2.3. Analytical methods

For each of our study species, we modelled evolutionary changes in sprouting probability in response to predicted local climate change. This involved the following steps, which are described in detail below: (i) analysis of the key vital rates in each population, including survival, sprouting, flowering and, in *Cypripedium*, fruiting, to model them as linear functions of density, weather and age or previous life history, (ii) using these functions to develop high-dimensionality population projection matrices, (iii) examination of demographic behaviour for evidence of density dependence, (iv) development and testing of climate-sensitive adaptive dynamics models to predict optimal sprouting probability, and (v) using these adaptive dynamics models to predict evolutionary trends in sprouting probability in response to predictions of future climate at the sites occupied by the species.

#### 2.3.1. Vital rate assessment

We used general linear mixed models (GLMMs) to explore the influences of selected ecological characteristics and climatic variables on all major vital rates and transition probabilities between different life states, including all size classes of flowering and non-flowering individuals, and vegetative dormancy, using the *lme4* package in R v. 3.2.2 [43,44]. The vital rates and probabilities estimated in our analysis were: survival probability from year *t* to year *t *+ 1 (binomial), sprouting probability in year *t *+ 1 conditional upon survival between years *t* and *t *+ 1 (binomial), probability of growth for every size class in year *t *+ 1 conditional upon both survival and sprouting (Poisson, with variance not significantly different from the mean in all three species), flowering probability in year *t *+ 1 conditional upon survival and sprouting in the same year (binomial), and number of flowers per individual in year *t* conditional upon sprouting and flowering in the same year (Poisson). In *Cypripedium*, fruiting probability of flowering individuals in year *t* conditional upon flowering (binomial), and number of fruits per individual in year *t* conditional upon flowering and fruiting in the same year (Poisson) were also estimated.

Each vital rate served as an independent response term in a GLMM global model that incorporated status across the two previous years, and climate in the current year. The following independent factors were used in all global models: size in year *t* (for *Cypripedium*, the measure of size was total number of sprouts; for *Ophrys* it was number of leaves per sprout), flowering status in year *t* (binomial: sprouts either flowered or they did not), and two weather variables, one representing temperature and the other representing precipitation at the sites occupied by each of the species. For *Cypripedium*, these variables were the total number of days with minimum temperatures below freezing in the winter before recording (termed ‘winter frost days’), and total annual precipitation in the preceding calendar year. For *Ophrys*, the variables were number of hours of sunlight in April and May, and total precipitation from the start of February to the end of May, in the year of recording. The choice of these climatic variables was based on previous analyses of the responses of these species to weather variables [38,39].

In previous analyses [28], we determined that estimates of costs of growth and reproduction were required across 3 years to accurately model the life history of *Cypripedium*. To incorporate longer-term individual history, we also included size and flowering status of individuals in year *t* − 1 in *Cypripedium* models. In *Ophrys*, we had no previous analyses to suggest the importance of long-term life history. Furthermore, the short lifespan of this species relative to the length of the observation period led us to test the impact of age rather than individual history. So, in *Ophrys*, we used time since first observation as a proxy for age from germination, instead of status (i.e. size and flowering status) in year *t* − 1 [38]. Because our two time-step approach to *Ophrys* yielded adaptive dynamics models consistent with observed sprouting patterns, we did not attempt to include individual history in any further mixed modelling exercises in this species. Finally, in *Cypripedium*, in models of fruiting probability and number of fruits, we replaced size in year *t* with the number of flowers produced by each sprout in year *t*, because more than 95% of flowering sprouts only had one flower. These models served as global, or most parametrized, models, and included all two-way interactions between fixed factors, with year as the sole random factor.

We constructed all possible reduced models from the global models. The model with the lowest AICc was typically used as the best-fit model for inference, except in cases where an equally parsimonious model (i.e. ΔAICc ≤ 2.0) had fewer parameters. Exhaustive model assessment was conducted in R using the *lme4* package [43] in conjunction with the *pdredge* function in package *MuMIn* [45], which allowed us to parallelize model selection using the *snow* package [46] and L. Aslett's pre-made Amazon EC2 R image [47].

#### 2.3.2. Matrix development

GLMMs were used to develop functions for all demographic processes required to create population projection matrices that could be used in both deterministic and stochastic simulations. The *Cypripedium* matrix models were ‘historical’ matrices, i.e. two-dimensional matrices of status across 3 years, with ‘stages’ for columns and rows corresponding to each pair of possible life-history stages in years *t* − 1 and *t*, and years *t* and *t *+ 1, respectively [48]. These models incorporate the historical life-history costs observed in our best-fit demographic GLMMs, which often opposed relationships between vital rates across 2 years. They also allowed the modelling of unique costs such as costs of growth (e.g. the cost to survival between years *t* and *t *+ 1 of high growth between years *t* − 1 and *t*), which have been noted both in this genus and in other herbaceous perennials [28,49]. In a given year, each plant occupied one of 19 mature states with anything from one to nine sprouts, either all vegetative or at least one of which was flowering, or it was completely dormant (electronic supplementary material, figure S1*a*). Transition probability values between life stages were given as

2.1 $$\begin{array}{rl} a_{D,ji} & =S_{ji}\times (1-P_{ji}), \end{array}$$

2.2 $$\begin{array}{rl} a_{k(V),ji} & =S_{ji}\times P_{ji}\times g_{k,ji}\times (1-F_{k,ji}) \end{array}$$

2.3 $$\begin{array}{rl} \text{and}\;\;a_{k(F),ji} & =S_{ji}\times P_{ji}\times g_{k,ji}\times F_{k,ji}, \end{array}$$

where *a_k,ji_* is the probability of transitioning to stage *k* in year *t *+ 1, given state *j* in year *t* and state *i* in year *t* − 1; *S_ji_* is the probability of surviving from year *t* to year *t *+ 1, given state *j* in year *t* and state *i* in year *t* − 1; *P_ji_* is the probability of sprouting in year *t *+ 1, given survival from year *t* and state *j* in year *t* and state *i* in year *t* − 1; *g_k,ji_* is the probability of growth to state *k* in year *t *+ 1, conditional upon survival to that time and given state *j* in year *t* and state *i* in year *t* − 1; and *F_k,ji_* is the probability of flowering in year *t *+ 1, conditional upon survival to that point and sprouting in that year, and given state *k* in year *t* + 1, state *j* in year *t* and state *i* in year *t* − 1. *D*, *V* and *F* refer to vegetative dormancy, vegetative (i.e. non-flowering) sprouting and flowering, respectively. *S_ji_*, *P_ji_*, *g_k,ji_* and *F_k,ji_* were typically functions of climate in addition to state in previous years, as given in the best-fit GLMM models of vital rates (electronic supplementary material, tables S1 and S2). For example, the probability of sprouting in *C. parviflorum* in year *t *+ 1 was given as

2.4 $$\begin{array}{r} P_{ji}=\text{logit}\left(\begin{array}{l} x_{0}+d_{spr}+(x_{dt32,t+1}\times dt32_{t+1})+(x_{tpcp,t+1}\times tpcp_{t+1})+(x_{siz,t}\times siz_{t})+(x_{flwyn,t}\times flwyn_{t})\\ \;+\;(x_{grw,t}\times grw_{t})+(x_{siz,t\times grw,t}\times siz_{t}\times grw_{t})+(x_{siz,t\times flwyn,t}\times siz_{t}\times flwyn_{t})\\ \;+\;(x_{grw,t\times dt32,t-1}\times grw_{t}\times dt32_{t-1})+(x_{grw,t\times tpcp,t-1}\times grw_{t}\times tpcp_{t-1}) \end{array}\right), \end{array}$$

where all *x*s refer to estimated coefficients from the model, including *x*_0_, the *y*-intercept; *d_spr_* refers to the deviation from the *y*-intercept used in adaptive dynamics simulations and is set to 0 otherwise (see Adaptive dynamics); *dt32_t_*_+1_ is the number of days with minimum temperatures less than or equal to 0°C in year *t *+ 1; *tpcp_t+1_* is the total annual precipitation in year *t*; *siz_t_* is size in year *t*; *flwyn_t_* refers to whether or not the individual flowered in year *t* (binomial) and *grw_t_* is the change in size between years *t* − 1 and *t* (electronic supplementary material, table S1).

The *Cypripedium* life cycle also included a minimum of 4 years in each of which an individual could occupy one of 10 juvenile stages (three protocorm stages, a new seedling stage and non-flowering, 1- and 2-year-old seedlings with 0, 1 or 2 sprouts), plus a dormant seed stage (electronic supplementary material, figure S1*a*). These juvenile stages were not monitored for this study, because these species are of conservation concern and monitoring these subterranean stages would cause considerable damage to the population and its habitat. Instead, these elements of the matrices were parametrized using data from other studies [50–53]. We tested the sensitivity of our results to different assumed numbers of seeds per fruit, percentage germination and probability of transition to seedlings, by increasing or decreasing each by 10% and repeating our adaptive dynamics analyses. These alterations did not appreciably change estimates, and therefore we do not present those results further. Altogether, these historical matrices had dimensionality of 395 × 395. Further details of the *Cypripedium* matrix model construction are provided by Shefferson *et al*. [28].

The *Ophrys* matrix model was an age × stage model with 10+ years of adult life (we modelled stasis in the adult stage corresponding to year 10 to allow for long lifespan), and 17 mature states per year of adult life (one to eight leaves, either flowering or vegetative, plus vegetative dormancy), in addition to a minimum of 2 years in three unmonitored juvenile stages (two protocorm stages and one seedling stage; electronic supplementary material, figure S1*b*), yielding a matrix with dimensionality of 173 × 173. Modelling involved the development of transition probabilities from vital rate functions based on the best-fit GLMM models of vital rates (electronic supplementary material, table S3), as in *Cypripedium*.

We developed deterministic and stochastic versions of both models, with both incorporating climatic variables. The stochastic models included random draws of values from the distributions of climatic variables, while the deterministic models incorporated only the mean climatic values. The stochastic models also incorporated a random year term in each estimated vital rate. This was drawn from the random year effect using the best-fit GLMMs for each term.

#### 2.3.3. Assessment of density dependence

Adaptive dynamics models assume that there is competition between genotypes. This requirement is generally met in life-history simulations by assuming the operation of negative density dependence in a key vital rate. Usually, only one key vital rate is chosen in order to create the most parsimonious model [54]. We used GLMMs to test all demographic rates and probabilities included in the matrices for conspecific density dependence (fixed factor) in all years (random factor), for the purpose of identifying a suitable negatively density-dependent parameter. Although transitions from germination and recruitment to the seedling stage are strongly density-dependent in many herbaceous perennials [54], we could not test for this in our species. Instead, we sought evidence of density dependence in the adult stages. The density of adult plants was estimated by placing a grid of 1 × 1 m over maps of each population and counting the number of individuals in each square. In *Ophrys*, sprouting individuals always consisted of a single sprout, whereas the number of sprouts differed between *Cypripedium* individuals. In *Cypripedium*, we used the number of individuals rather than number of sprouts per unit area to assess density.

#### 2.3.4. Adaptive dynamics

The matrices described above were used in adaptive dynamics simulations to determine the optimal sprouting probability in each species. We treated the *y*-intercept in the GLMM for sprouting as the evolving trait in our analysis, both because the *y*-intercept has been used as the target parameter of evolution in previous studies [32,54] and because there is currently insufficient understanding of sprouting to allow the use of a more fine-scale, mechanistic sprouting trait. This intercept is a constant in the binomial equation determining sprouting probability, and can be positive or negative. It represents the intrinsic tendency to sprout, with higher/lower values indicating increasing/decreasing sprouting probability, regardless of plant state or weather. We developed resident and invader matrices using set values of climate-related variables, and used deviations from the *y*-intercept in the GLMM as inputs in these matrices. Our method is based on a similar approach for determining optimal flowering frequency in monocarpic perennials [54].

Simulations of each species' adaptive dynamics began with a single plant with a deviation in its probability of sprouting from the estimated *y*-intercept value. These deviations produced a range of sprouting probabilities from very infrequent (as low as approx. 20% in some conditions) to near-certain probabilities of sprouting. The plant was assumed to multiply clonally until the population reached a plateau density. This density was obtained by optimizing *α* and *β* in a Ricker function that served to modify a selected density-dependent vital rate in each population. (Although our study species are primarily sexual rather than clonal, other analyses have demonstrated the robustness of this approach in retrospective analyses [22,28,54].) The plateau population density approximated that observed during monitoring.

For simplicity, we assumed that there was only one negatively density-dependent demographic parameter in each population. This parameter was chosen by testing whether its density dependence function yielded a realistic plateau density. Because we sought to develop a parsimonious model, we did not incorporate all density-dependent rates, but focused on finding a single rate that appeared most likely to respond to competition between conspecifics. In each case, a process of iteration and testing against all negatively density-dependent vital rates was used to select a rate that yielded asymptotic population growth (most did not). Following analyses showing that no other demographic parameter yielded realistic density plateaus, sprouting probability was the selected parameter for both *Cypripedium* species. In *Ophrys*, the selected parameter was adult survival probability.

Initial model optimization assumed a sprouting *y*-intercept deviation of 0. Once a plateau population density was reached, a single invading mutant with either the same or a different sprouting *y*-intercept was introduced into the population, and allowed to multiply clonally. The average growth rate of the mutant proportion of the population over 100 annual time steps was used as a measure of the relative fitness of the mutant. This process was repeated for pairs of resident and mutant individuals, each with a wide range of sprouting probabilities, to create a fitness landscape showing the conditions under which mutant sprouting strategies would spread, go extinct or remain at equilibrium with resident strategies (termed pairwise invasibility analysis [55]). Evolutionarily stable strategies in such analyses are areas of the fitness landscape to which the trait evolves over time, and may be local or general, and stable or unstable.

We developed both deterministic and stochastic versions of these models, with mean values of climate variables recorded over the monitoring periods as input. The stochastic models differed from the deterministic models by including random draws with replacement of years, each of which was associated with its own random year intercept from the associated GLMM, and values for climatic variables, which were selected randomly from the distributions of climatic variables at each site. We used the deterministic models in evolutionary prediction both because the deterministic models yielded sprouting *y*-intercepts that did not differ significantly from those estimated in the original GLMMs, and because the stochastic models were less parsimonious, and involved greater uncertainty.

#### 2.3.5. Predicting optimal sprouting patterns in response to climate change

After determining the optimal sprouting *y*-intercept for the climatic conditions recorded during the years of monitoring, we repeated the adaptive dynamics simulations, as described above, using predicted values for climate variables for the years 2075–2099 at each of the study locations. The model used, MRI-AGCM3.2S, predicts future climate at a spatial resolution of 20 × 20 km, and at a temporal resolution of 1 day [33,56]. It has been validated using real climate data for 1979–2003 in the greater Pacific region [33,56], and used to predict changes in population growth rates in some desert plant species caused by changes in survival and other demographic parameters associated with climate change [16]. Because the simulated data in this model only cover the periods 1979–2003 and 2075–2099, we also used simulated climate values from MRI-AGCM3.2H, a companion model that predicts climate at a spatial resolution of 60 × 60 km for every day from 1 January 1979 to 31 December 2099 [34]. We also obtained data from both models for surface-level shortwave radiation received per month (W m^−2^) at the *Ophrys* site. This was used to estimate the number of hours of sunlight expected per day throughout the simulation period.

In addition to simulated climate data, actual climate data for the *Cypripedium* site were obtained from the National Centers for Environmental Information (www.ncdc.noaa.gov) and, for the *Ophrys* site, from the nearest Meteorological Office weather station, at Eastbourne, East Sussex, UK [38]. To make these data compatible with the projected climate data, we also developed functions predicting number of freezing days at the *Cypripedium* site and number of sunlight hours from February to May at the *Ophrys* site, as functions of weather variables (generally daily or monthly temperature, and precipitation totals) common to both the actual and modelled climate datasets. Sunlight hours were estimated from projected surface-level shortwave radiation using the Suehrcke method, with constants for southern England supplied from the literature [57,58].

Initially, we developed a full adaptive dynamics model for each population using the 2075–2099 predicted climate data from the MRI-AGCM3.2S model. We also assessed evolutionary optima from 1979 to 2099 using the predicted climate from MRI-AGCM3.2H. Rather than using each year's climate as the optimization input, we used the mean of climate values for each year and the preceding 25 years. This allowed the optimization procedure to more realistically reflect our use of the mean climate over 21–32 years of actual observation to determine the optimum sprouting tendency, and to buffer predictions from exhibiting large fluctuations from year to year. Each year, from the end of monitoring until 2099, thus had its own associated optimum value for the *y*-intercept of sprouting, reflecting climatic conditions over the previous 25 years. Rather than representing values that will actually occur in the future, these sprouting tendencies should be interpreted as optima towards which natural selection will direct our target trait. Standard errors for these optimal *y*-intercepts were estimated using 1000 hierarchical, non-parametric bootstraps of randomly selected individuals from the original demographic datasets.

Because simulated climate data did not match observed climate data perfectly over the calibration period (1979–2003), we corrected simulated data by the difference in mean climate values between observed and simulated data during the years in which they overlapped. To assess the effect of this correction against the projected validation data, we also re-ran the analysis without this correction. Qualitatively similar results were obtained for *C. candidum* and *Ophrys*. The uncorrected *C. parviflorum* model failed to converge.

Finally, we assessed the impacts of predicted evolutionary trends on population growth rate in all three species. First, we estimated population growth rate for each year from 1979 to 2099 using matrix models parametrized with the climate projected from model MRI-AGCM3.2H for that year, under scenarios under which sprouting either did or did not evolve. Here, population growth rate was estimated as deterministic *λ*, or the dominant eigenvalue for the annual matrix, and optimal *y*-intercept values were estimated with each 25-year running mean of weather variables. Next, we looked at the effect of evolution in sprouting on the population growth rate via life table response analysis. To do this, we compared projection matrices in which sprouting evolved, against matrices in which it did not, over the period 2075–2099. We performed this analysis using the *popbio* package [59] in R v. 3.2.2 [44].

## 3. Results

### 3.1. Population and climatic trends

Above-ground population size in both *Cypripedium* populations was roughly stable at the beginning of the study, but declined towards 2010. The number of emergent plants in the *Ophrys* population was low until 1991, after which it increased nearly sixfold, and then began a slow decline to the end of the study, in 2006. Estimated numbers of living juveniles + adults (not including seedlings) ranged between years from 330 to 575 in *C. parviflorum*, from 46 to 71 in *C. candidum*, and from 41 to 657 in *Ophrys*. Estimates of annual deterministic *λ* ranged from 0.971 to 1.138 (mean ± 1 s.e. = 1.056 ± 0.011) in *C. parviflorum*, from 0.919 to 1.144 (mean ± 1 s.e. = 1.062 ± 0.013) in *C. candidum*, and from 0.869 to 1.123 (mean ±1 s.e. = 0.992 ± 0.015) in *Ophrys*.

**Figure 1.**
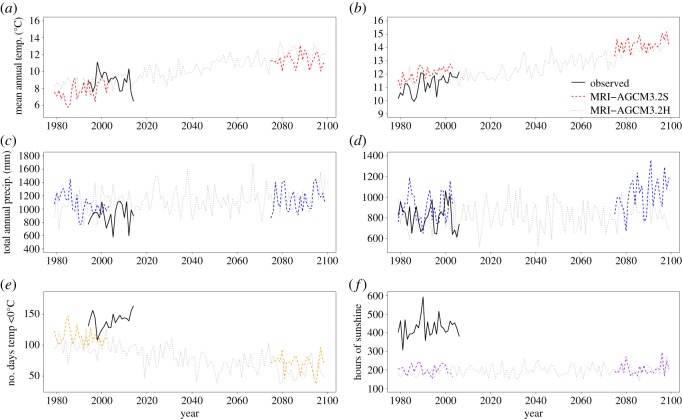
Observed and modelled climate at Gavin Prairie Nature Preserve (*a*,*c*,*e*) and Castle Hill National Nature Reserve (*b*,*d*,*f*). Observed data were obtained from the National Centers for Environmental Information (NOAA, USA) and the Meteorological Office weather station, at Eastbourne, East Sussex, UK. Modelled data were obtained from models MRI-AGCM3.2S (20 × 20 km resolution) and MRI-AGCM3.2H (60 × 60 km resolution). Panels show actual and modelled values for mean annual temperature (*a*,*b*), total annual precipitation (*c*,*d*), annual number of days with minimum temperatures below freezing (winter frost days, Gavin Prairie only, *e*), and hours of sunshine in April and May (Castle Hill only, *f*). Values for the latter two variables are presented because their influence on demographic parameters was assessed using mixed models.

**Figure 2.**
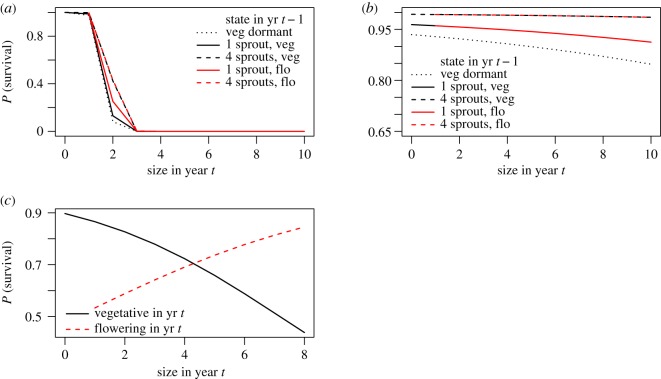
Life-history costs of growth to survival in *Cypripedium parviflorum* (*a*), *C. candidum* (*b*) and *Ophrys sphegodes* (*c*). Survival decreased with size in both *Cypripedium* species and in vegetative *Ophrys*, but increased with size in flowering *Ophrys*. In all cases, *P* (survival) refers to the probability of surviving from year *t* to year *t *+ 1. Plotted lines in (*a*) and (*b*) refer to different states in year *t *− 1, while plotted lines in (*c*) refer to different states in year *t*.

Simulations of climate until 2099 predicted warming at both study sites. At Gavin Prairie, model MRI-AGCM3.2S predicted that mean annual temperature would increase from 7.7 ± 0.2°C to 11.4 ± 0.2°C (mean ± 1 s.e.; *p* < 0.0001), and that precipitation would not change significantly (1067.9 ± 31.1 mm in 1979–2003, and 1143.0 ± 35.8 mm in 2075–2099, *p* = 0.173; figure 1*a*,*c*). At Castle Hill, temperature was predicted to increase from 12.0 ± 0.1°C to 14.2 ± 0.1°C (*p* < 0.0001), and precipitation from 903.0 ± 28.8 mm to 1023.3 ± 33.8 mm (*p* = 0.009; figure 1*b*,*d*). The mean number of freezing days per annum at Gavin Prairie was predicted to decrease from 112.8 ± 2.6 to 70.2 ± 2.7, *p* < 0.0001; figure 1*e*), while the number of hours of sunshine in spring at Castle Hill was predicted to remain similar to its current level (201.5 ± 5.2 in 1979–2003 versus 202.8 ± 6.4 in 2075–2099, *p* = 0.852; figure 1*f*). Observed climate data matched the distributions of simulated climate data except for number of hours of sunshine at Castle Hill, where observed values were approximately twice the simulated mean (424.7 ± 9.6 versus 201.5 ± 5.19, *p* < 0.0001; figure 2*f*).

### 3.2. Modelling demographic parameters

Vital rates and probabilities were strongly driven by size and flowering status. Best-fit models and equally parsimonious models with fewer parameters for most vital rates in all species typically included size and flowering status in year *t* as a predictor variable (electronic supplementary material, tables S4–S6). Individual history had a significant effect on all vital rates apart from juvenile sprouting in *C. parviflorum* (electronic supplementary material, tables S1 and S4), and flowering probability, fruiting probability, number of fruits and juvenile sprouting in *C. candidum* (electronic supplementary material, tables S2 and S5). In *Ophrys*, age since first observation affected survival, but did not affect other vital rates (electronic supplementary material, tables S3 and S6).

Sprouting was associated with costs to survival in all three species. In *C. parviflorum*, probability of survival was highest for plants that remained small or dormant between years, and for plants that fluctuated between large and small/dormant (figure 2*a*). Juvenile *C. parviflorum* plants exhibited lower survival with larger size (electronic supplementary material, table S1). In *C. candidum*, survival was highest in consistently large plants, but was also high in plants that fluctuated between vegetative dormancy and small size, and in plants that stayed small or dormant (figure 2*b*). In *Ophrys*, the highest survival occurred when plants remained dormant. Moreover, survival was lower for larger non-flowering, emergent plants, but in flowering plants there was a positive relationship between size and survival to the subsequent year (figure 2*c*).

**Figure 3.**
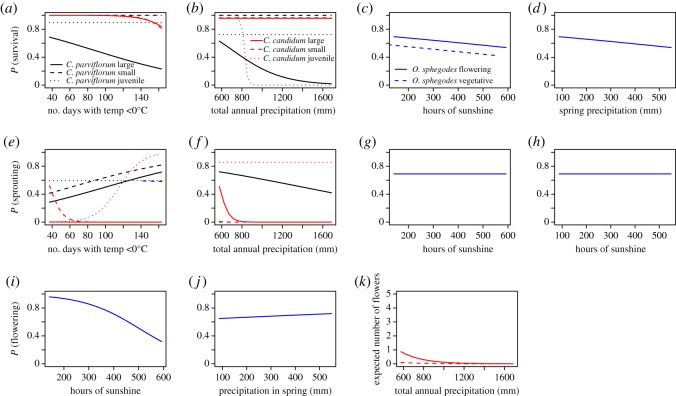
Relationships between demographic parameters and climate in *Cypripedium parviflorum* (*a*,*b*,*e*,*f*), *C. candidum* (*a*,*b*,*e*,*f*,*k*) and *Ophrys sphegodes* (*c*,*d*,*g*,*h*,*i*,*j*). Survival was typically negatively correlated with investigated climatic variables, while sprouting had mixed correlations with climate variables in all three species. Reproductive traits were typically uncorrelated with climate variables, except for the probability of flowering in *Ophrys* and with number of flowers produced in *C. candidum*. Parameters that showed significant linear correlations with climate variables are shown, except for sprouting, where all relationships are shown. Because relationships with climate are affected by size in all species, we present relationships assuming representative sizes, chosen in each case to show the variability in the demographic parameter illustrated.

Climate strongly influenced many vital rates. In *C. parviflorum*, adult survival and sprouting were functions of number of winter frost days and annual precipitation (figure 3*a*,*b*,*e*,*f*). In *C. candidum*, adult sprouting was a function of both of these climatic variables, whereas survival was only a function of winter frost days (figure 3*a*,*b*,*e*,*f*; electronic supplementary material, tables S1 and S2). In *Ophrys*, adult survival was negatively correlated with both number of hours of sunshine and spring precipitation (figure 3*c*,*d*), although this typically negative relationship was driven by interactions between size, flowering and climate (electronic supplementary material, table S3). Adult sprouting was not correlated with climatic variables (figure 3*g*,*h*; electronic supplementary material, table S3). Juvenile survival and sprouting were not functions of climate in *C. parviflorum* (electronic supplementary material, figure S3*a*,*b*,*e*,*f* and table S1). However, in *C. candidum*, juvenile survival decreased with increasing annual precipitation (figure 3*b*; electronic supplementary material, table S2), and, due to a strong interaction with size, juvenile sprouting increased with increasing number of winter frost days (figure 3*e*; electronic supplementary material, table S2). Reproduction was less sensitive to climate in all species. In *C. parviflorum*, climatic variables did not affect the probabilities of flowering or fruiting or the number of flowers or fruits produced. In *C. candidum*, the number of flowers produced by flowering sprouts decreased with precipitation, while other vital rates were not affected (figure 3*k*; electronic supplementary material, table S2). Finally, in *Ophrys*, the probability of flowering decreased with hours of sunshine and increased with precipitation (figure 3*i*,*j*; electronic supplementary material, table S3).

### 3.3. Density dependence

Survival was positively density dependent in both *Cypripedium* species, but negatively density dependent in *Ophrys* (electronic supplementary material, figure S2*a*). Sprouting probability was negatively density dependent in *C. parviflorum* juveniles and adults, and in *C. candidum* adults, but positively density dependent in *Ophrys* and in *C. candidum* juveniles (electronic supplementary material, figure S2*b*). Flowering probability was typically negatively density dependent, except in adult *Ophrys* (electronic supplementary material, figure S2*c*), whereas number of flowers produced was generally positively density dependent, except for *Ophrys* plants recorded for the first time, in which it was negatively density dependent (electronic supplementary material, figure S2*d*). Finally, fruiting probability and the number of fruits produced were positively density dependent in both *Cypripedium* species (electronic supplementary material, figure S2*e*,*f*).

### 3.4. Adaptive dynamics models and evolutionary predictions

Adaptive dynamics models predicted that observed *y*-intercepts for sprouting probability were evolutionarily stable strategies. The optimal deviation on the *y*-intercept in the mixed model predicting sprouting probability was 0.153 ± 0.168 (mean ± 1 s.e.), −0.131 ± 0.449 and 0.844 ± 0.451, for *C. parviflorum, C. candidum* and *Ophrys*, respectively (electronic supplementary material, figure S3*a*–*c*). These are additive deviations from the original *y*-intercepts estimated in our best-fit GLMMs (*C. parviflorum*: −2.065 ± 2.007, *C. candidum*: 0.410 ± 2.052, *O. sphegodes*: 0.237 ± 0.200; electronic supplementary material, tables S1–S3). In all cases, the confidence interval for the optimal deviation included 0, and therefore the models successfully predicted the sprouting probabilities observed over the monitoring periods. When the uncorrected portion of the simulated climate data that overlapped with field data was used as input, the optimal sprouting *y*-intercept deviations were 0.500, 0.431 and 0.630 for *C. parviflorum*, *C. candidum* and *Ophrys*, respectively.

**Figure 4.**
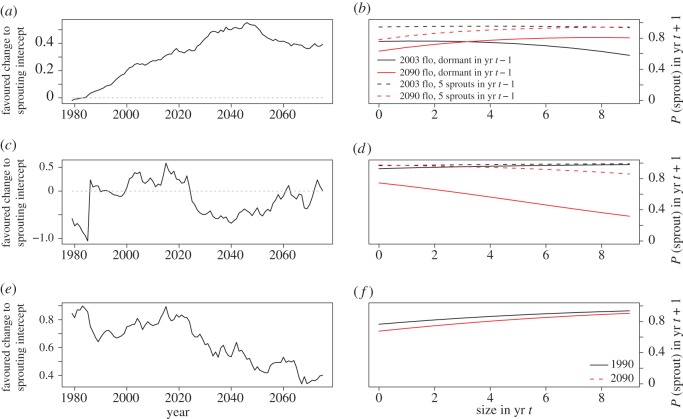
The evolution of sprouting probability in response to climate change in *Cypripedium parviflorum* (*a*,*b*), *C. candidum* (*c*,*d*) and *Ophrys sphegodes* (*e*,*f*). Panels on the left show the optimal deviation in the *y*-intercept in the mixed model of sprouting in each species across time (scale differs between plots to enable temporal trends to be visualized more easily). Here, the optimal deviation represents the shift in the *y*-intercept favoured by natural selection, where the *y*-intercept in the linear model of sprouting represents an intrinsic tendency to sprout regardless of environmental conditions or current status/size. Panels on the right show the associated sprouting probabilities in year *t *+ 1 during the monitoring periods (black) versus the predicted values in 2075–2099 (red), as dependent on size in year *t*, flowering status in year *t* (*Ophrys*) and size in year *t *− 1 (both *Cypripedium* species). Dashed grey lines in panels (*a,c*) mark a sprouting deviation of 0.

Although predicted climate trends are similar at both study sites, the models predicted different evolutionary trajectories for sprouting probability in the three species. In *C. parviflorum*, predicted climate change was associated with generally increasing sprouting probability, with the optimal value stabilizing by 2035 (figure 4*a*,*b*). In *C. candidum*, the models predicted an initial decrease in sprouting probability, followed by a large increase before returning to a level close to its current value (figure 4*c*,*d*). In *Ophrys*, the models predicted decreasing sprouting probability until 2050, followed by relatively stable sprouting behaviour (figure 4*e*,*f*). The optimal deviations on sprouting predicted for 2075–2099 were 0.831 ± 0.684, −0.408 ± 0.042 and 0.435 ± 0.302 for *C. parviflorum*, *C. candidum* and *Ophrys*, respectively (electronic supplementary material, figure S3*d*–*f*).

Evolution in sprouting probability appeared to be driven by interactions between survival, sprouting, size and climate. The predicted changes in climate had contrasting effects on the growth probability distributions in the two *Cypripedium* species, with warmer, drier years resulting in greater growth in *C. parviflorum* (electronic supplementary material, table S1), but lower growth, or even shrinkage in *C. candidum* (electronic supplementary material, table S2). In both species, higher temperature and lower precipitation drive higher survival (figure 3*a*,*b*,*e*,*f*), but interact with growth and size resulting in lower survival when growth is high or plants are large (figure 2*a*,*b*; electronic supplementary material, tables S1 and S2). Flowering and fruiting appear relatively unaffected by climate in these two species, except that more flowers are produced per individual in *C. candidum* in drier years (figure 3*k*). In *Ophrys*, more sunshine and greater precipitation are associated with greater growth and lower survival (figure 3*c*,*d*). Sprouting probability appears unaffected by climatic variables (figure 3*g*,*h*), although the probability of flowering decreases with increasing sunshine and increases with increasing precipitation (figure 3*g*,*h*; electronic supplementary material, table S3).

### 3.5. Impacts of evolution on population growth rate

Deterministic population growth rates (*λ*) estimated until 2099 using models with and without evolution in sprouting, suggested decreasing *λ* in *C. parviflorum*, increasing *λ* in *C. candidum*, and fluctuating *λ* in *Ophrys* (electronic supplementary material, figure S4). In all cases, evolution in sprouting caused marginal increases in mean values of *λ* (electronic supplementary material, figure S4). Projected differences in *λ* due to evolution were driven primarily by altered transitional values in plants remaining in the vegetative or dormant state in *C. parviflorum* (electronic supplementary material, figure S5) and *C. candidum* (electronic supplementary material, figure S6), and by stasis in dormant or small individuals in *Ophrys* (electronic supplementary material, figure S7). Particularly, altered stasis in dormancy among typically dormant individuals, and altered probabilities of transition to flowering after two vegetative years, had positive effects on *λ*, while altered growth to flowering among typically dormant individuals, and altered shrinkage after two vegetative years had a negative effect on *λ* in *C. parviflorum* (electronic supplementary material, figure S5). Patterns were similar in *C. candidum*, except that altered shrinkage transitions among consistently vegetative individuals tended to increase *λ* (electronic supplementary material, figure S6). Finally, in *Ophrys*, growth from dormancy had a positive effect on *λ*, particularly when it involved transitions to flowering stages (electronic supplementary material, figure S7). By contrast, stasis in dormancy reduced *λ* the most, followed by shrinkage to small vegetative stages (electronic supplementary material, figure S7).

## 4. Discussion

Our models predict that the probability of sprouting will evolve in different directions over time in the three study species. Even at the same site, the congeneric *C. parviflorum* and *C. candidum* were predicted to evolve differently, with natural selection favouring increasing sprouting probability throughout the next 80 years in the former species, and fluctuating sprouting probability around the current value in the latter (figure 4*a*,*c*). In contrast with both of these predictions, *Ophrys* is forecast to evolve greater dormancy over the same period (figure 4*e*). These differences are especially striking given the similarities in predicted climatic trends at the two sites occupied by these species.

The differing predictions largely contradict our original hypothesis that climate change will result in the evolution of greater vegetative dormancy. Our hypothesis was based on the assumptions that vegetative dormancy is a response to stress, and that climate change is stressful. The former assumption is rooted in *in situ* experiments showing decreased sprouting after defoliation in *Cypripedium calceolus* and *Cephalanthera longifolia* [29], and after experimental pollination in *Cypripedium acaule* [60], and in the hypothesis that sprouting itself may be costly, despite being necessary [26,28]. The assumption that climate change is stressful is based on empirical data suggesting that many natural plant populations are currently experiencing stress, as illustrated by decreased population growth rates and increased extinction risks [61,62]. However, in the three study species, higher temperature was often associated with higher survival, while higher precipitation often had the opposite effect (figure 3*a*–*d*; electronic supplementary material, tables S1–S3). Furthermore, population growth rate is forecast to decline in *C. parviflorum*, the species which is predicted to evolve greater sprouting (figure 4*a*,*b*; electronic supplementary material, figure S4*a*), while *Ophrys* is predicted to evolve greater dormancy, while exhibiting an increasing population growth rate (figure 4*e*,*f*; electronic supplementary material, figure S4c). In *Ophrys*, annual recruitment and mortality are both positively correlated with the previous year's temperature, whereas the proportion of plants that flower shows the opposite pattern [38]. Recent northwards range expansion of this species in the UK [37] suggests that the overall effect of contemporary climate change on *Ophrys* is beneficial, despite being associated with decreased sprouting. Indeed, although survival and flowering are expected to decline with increased hours of sunshine in *Ophrys* (figure 3), the number of hours of sunshine is not expected to change substantially before 2099 (figure 1). As climate is predicted to become warmer and wetter at both study sites, these results cast doubt on the notion that climate change will be a major source of stress for these species over the next century.

Although our models predict contrasting trends in the evolution of sprouting, we also note a similar life-history context to this trait in all three species. In particular, although sprouting exhibits a range of relationships with size and flowering status, growth exerts costs upon survival in all three species, at least in juvenile and vegetative plants (figure 2). These may be physiological or energetic costs of building above-ground structures, which use stored reserves beyond critical levels necessary for coping with difficult future conditions [28,49]. This finding provides strong evidence that vegetative dormancy is an adaptive trait, rather than a bet-hedging trait, at least in these species.

The predictions of our models reflect a changing adaptive landscape of sprouting probability in relation to other demographic parameters and in response to predicted changes in climate. Sprouting strongly influences fitness for several reasons. First, it is essential for sexual reproduction, as flowers cannot form and pollination cannot occur without above-ground growth. Second, sprouting can have a strong impact upon survival, particularly if it increases mortality by consuming resources that could have sustained the plant against adverse conditions or greater herbivory as a consequence of climate change [28]. Third, sprouting may exert a cost upon future growth and sprouting, making one or both less likely in future [60]. Finally, sprouting may exert a cost on reproduction. As fitness is a function of sprouting and dormancy [24,63], and as demographic parameters often vary considerably in response to climate [16,64], climate change is likely to alter the context of relationships between sprouting and fitness. For example, in *C. parviflorum*, higher temperature and precipitation drive higher sprouting probabilities and larger size, and are associated with lower mortality, resulting in selection for increased probability of sprouting. However, growth to larger sizes also results in higher mortality, lower future sprouting probability, and a higher probability of shrinkage, the latter two of which are more pronounced with higher precipitation (electronic supplementary material, table S1). Growth also leads to a higher probability of flowering in *C. parviflorum*. This is associated with decreased survival in the following year, but increased survival in the year after that. In *C. candidum*, the life-history context surrounding vegetative dormancy is simpler, because plants have higher survival when they are dormant or small, or large and flowering. Finally, *Ophrys* evolves a lower probability of sprouting under warmer, wetter conditions, because of strong positive relationships between precipitation and growth, and mortality and size, and by a strong negative impact of reproduction on survival.

Our predictions are dependent upon these populations being able to evolve quickly. The speed of evolution in response to climate change may be limited by existing genetic variation, population size, gene flow and the strength of selection [65]. It may also be influenced by the extent to which environmental factors and traits impose constraints and lags on the trait of interest. Although we do not have data on variation for sprouting potential within the study populations, many demographic parameters are known to have high genetic variance [66], and different parameters often exhibit strong genotype × environment interactions [67], which can preserve genetic variation if the relative fitness of genotypes changes over time. Although orchid populations are often small, increasing the possibility of genetic drift, natural selection can still act strongly and proceed more quickly in smaller populations in some contexts [68]. In addition, our predictions assume that relationships between vital rates, and between vital rates and climate variables, will not change over time, and particularly that they will not evolve in ways that indirectly impact sprouting.

Adaptive evolution in demographic parameters such as sprouting probability might at least partially counteract any increase in the risk of extinction associated with climate change. Evolutionary rescue occurs when a threatened population avoids extinction through adaptation [69]. Although natural selection can reduce population size [70], adaptive evolution may counter the forces moving populations towards extinction by favouring genotypes that increase population growth [71]. Given that changes in population dynamics in response to climate change can now be predicted [16,22], the integration of evolutionary change into existing methods for predicting population trajectories may allow conservation biologists and managers to assess the potential for evolutionary rescue.

The primary challenge in predicting the impacts of climate change on population or species viability may not be to quantify evolutionary changes in traits of interest to the evolutionary biologist, but to identify the traits that are most likely to evolve in response to climate change. Addressing this challenge is important, because the evolution of any trait depends on many factors, including its genetic basis, its relationships with other traits, population size and spatial distribution [72]. In this regard, the development of a modelling framework to predict the simultaneous evolution of multiple traits, particularly under genetic and regulatory linkage, is an important challenge. Genetic correlations are a major test for evolutionary prediction, because they can lead to evolution in traits that are not expected to evolve, they can speed up or slow down evolution in traits of interest, and they can even drive the evolution of seemingly suboptimal or maladaptive traits [73,74]. Such correlations may also behave as trade-offs that are potentially capable of evolution in response to climate change, and may in some cases act across years via lagged effects. Although we did not allow evolution of trade-offs in our models, all trade-offs might all be capable of evolution, potentially in unexpected directions [75,76].

Predicting the evolutionary responses of species will become increasingly important as the impacts of climate change intensify. Evolution can be rapid [77], and it can influence ecological processes at the population, community and ecosystem scales [21,78]. We have used a relatively simple approach to predict evolutionary trends in just one trait. Only continued monitoring until 2099 will demonstrate whether our predictions reflect realistic evolutionary trajectories. Although analysis of the impacts of evolution using more traits, and of its impact on communities and on ecosystems, poses a greater challenge, the difficulties are certainly surmountable. The insights gained from this approach may strengthen approaches to management by taking into account more realistic predictions of the consequences of rapid climate change.

## Supplementary Material

Figure S1. Life cycles of study species

## Supplementary Material

Figure S2. Density dependence in vital rates

## Supplementary Material

Figure S3. Pairwise invasability plots.

## Supplementary Material

Figure S4. Projected deterministic growth rates through 2099

## Supplementary Material

Figure S5. Life table response experiment analysis for C. parviflorum

## Supplementary Material

Figure S6. Life table response experiment analysis for C. candidum

## Supplementary Material

Figure S7. Life table response experiment analysis for Ophrys sphegodes

## Supplementary Material

Table S1. Best-fit parameters for C. parviflorum

## Supplementary Material

Table S2. Best-fit parameters for C.candidum

## Supplementary Material

Table S3. Best-fit parameters for O. sphegodes

## Supplementary Material

Table S4. Model selection table for C. parviforum

## Supplementary Material

Table S5. Model selection table for C. candidum

## Supplementary Material

Table S6. Model selection table for O. sphegodes

## Data accessibility

Demographic data used in this study is available through Dryad (doi:10.5061/dryad.g3fn8) [79].
