# Supplementary figures and images for: Predicting evolution in response to climate change: the example of sprouting probability in three dormancy-prone orchid species
Source: R Soc Open Sci. 2017 Jan 18;4(1):160647. doi: 10.1098/rsos.160647 (PMC5319331; doi:10.1098/rsos.160647)

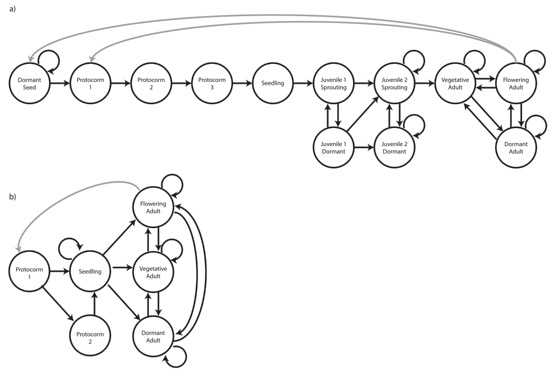

Supplement: Figure S1. Life cycles of study species [file rsos160647supp1.jpg]
