# Supplementary material for: Predicting evolution in response to climate change: the example of sprouting probability in three dormancy-prone orchid species
Source: R Soc Open Sci. 2017 Jan 18;4(1):160647. doi: 10.1098/rsos.160647 (PMC5319331; doi:10.1098/rsos.160647)
Supplement: Table S1. Best-fit parameters for C. parviflorum [file rsos160647supp8.docx]

**Table S1.** Estimates of the effects of factors on demographic parameters in a population of *Cypripedium parviflorum*, monitored for 21 years at Gavin Prairie Nature Preserve, Lake County, Illinois, USA. Fixed factors include size in year *t* (*Siz*_t_), growth between years *t*-1 and *t* (*Grw*_t_, given as *Siz*_t_-*Siz*_t-1_), flowering status in years *t*-1 and *t* (*Flw*_yn,t-1_ and *Flw*_yn,t_), number of flowers in year *t* (*Flw*_t_), total annual precipitation in year *t* and year *t*+1 (*TPCP*_t_ and *TPCP*_t+1_, respectively), and the number of days with temperatures below 0°C in year *t* and year *t*+1 (*DT32*_t_ and *DT32*_t+1_, respectively). Size was measured as the number of aboveground sprouts. Year was included as a random effect in all models. As seedlings could not be tracked longitudinally, no models are presented for that stage. Mixed model analysis was conducted using function *glmer* in package *lme4* in *R* 3.2.2 ([Bates, Maechler & Bolker 2012](#_ENREF_1); [R Core Team 2012](#_ENREF_22)). Estimates are derived from the model with the lowest AICc unless noted, in which case they are derived from an equally parsimonious model with fewer parameters.

| *C. parviflorum* models | | | |
| --- | --- | --- | --- |
| Effects | Estimate | SE | *P* ≤ |
| Adult survival probability | |  |  |
| Intercept | 3.875 | 1.157 | 0.001 |
| *DT32*_t+1_ | -0.016 | 0.007 | 0.024 |
| *TPCP*_t+1_ | 0.009 | 0.007 | 0.158 |
| *Siz*_t_ | 0.316 | 0.259 | 0.223 |
| *Grw*_t_ | -2.017 | 0.384 | 0.0001 |
| *Flw*_yn_,_t_ | -0.574 | 0.173 | 0.001 |
| *Flw*_yn_,_t-1_ | 0.156 | 0.129 | 0.226 |
| *Siz*_t_ *× TPCP*_t+1_ | -0.007 | 0.003 | 0.016 |
| *Siz*_t_ *× Grw*_t_ | 0.108 | 0.023 | 0.0001 |
| *Siz*_t_ *× Flw*_yn_,_t_ | 0.484 | 0.091 | 0.0001 |
| *Grw*_t_ *× DT32*_t+1_ | 0.009 | 0.003 | 0.001 |
| *Grw*_t_ *× Flw*_yn_,_t-1_ | 0.259 | 0.098 | 0.009 |
|  |  |  |  |
| Adult sprouting probability | |  |  |
| Intercept | -2.065 | 2.007 | 0.305 |
| *DT32*_t+1_ | 0.022 | 0.013 | 0.079 |
| *TPCP*_t+1_ | -0.009 | 0.010 | 0.370 |
| *Siz*_t_ | 0.639 | 0.052 | 0.0001 |
| *Grw*_t_ | -0.097 | 0.274 | 0.723 |
| *Flw*_yn_,_t_ | 0.484 | 0.126 | 0.0001 |
| *Siz*_t_ *× Grw*_t_ | -0.015 | 0.007 | 0.047 |
| *Siz*_t_ *× Flw*_yn_,_t_ | -0.263 | 0.073 | 0.0003 |
| *Grw*_t_ *× DT32*_t+1_ | -0.004 | 0.002 | 0.026 |
| *Grw*_t_ *× TPCP*_t+1_ | 0.004 | 0.002 | 0.026 |
|  |  |  |  |
| Adult growth (*Siz*_t+1_) |  |  |  |
| Intercept | 0.382 | 0.142 | 0.007 |
| *TPCP*_t+1_ | -0.002 | 0.002 | 0.114 |
| *Siz*_t_ | -0.023 | 0.026 | 0.680 |
| *Grw*_t_ | -0.057 | 0.017 | 0.001 |
| *Flw*_yn_,_t_ | 0.293 | 0.182 | 0.107 |
| *Flw*_yn_,_t-1_ | 0.036 | 0.031 | 0.247 |
| *Siz*_t_ *× TPCP*_t+1_ | 0.002 | 0.001 | 0.0001 |
| *Siz*_t_ *× Grw*_t_ | -0.006 | 0.002 | 0.001 |
| *Siz*_t_ *× Flw*_yn_,_t_ | 0.057 | 0.023 | 0.012 |
| *Grw*_t_ *× Flw*_yn_,_t_ | 0.043 | 0.017 | 0.012 |
| *Grw*_t_ *× Flw*_yn_,_t-1_ | -0.056 | 0.015 | 0.0003 |
| *Flw*_yn_,_t_ *× TPCP*_t+1_ | -0.004 | 0.002 | 0.045 |
|  |  |  |  |
| Flowering probability |  |  |  |
| Intercept | -1.297 | 0.162 | 0.0001 |
| *Siz*_t_ | 0.210 | 0.045 | 0.0001 |
| *Grw*_t_ | 0.091 | 0.050 | 0.066 |
| *Flw*_yn_,_t_ | 1.123 | 0.088 | 0.0001 |
| *Flw*_yn_,_t-1_ | 0.930 | 0.092 | 0.0001 |
| *Siz*_t_ *× Grw*_t_ | -0.025 | 0.008 | 0.002 |
| *Grw*_t_ *× Flw*_yn_,_t-1_ | -0.139 | 0.057 | 0.016 |
|  |  |  |  |
| Flowering quantity |  |  |  |
| Intercept | -0.241 | 0.042 | 0.0001 |
| *Siz*_t_ | 0.228 | 0.013 | 0.0001 |
| *Grw*_t_ | 0.082 | 0.021 | 0.0001 |
| *Flw*_yn_,_t-1_ | 0.183 | 0.048 | 0.001 |
| *Siz*_t_ *× Grw*_t_ | -0.010 | 0.002 | 0.0001 |
| *Grw*_t_ *× Flw*_yn_,_t-1_ | -0.053 | 0.021 | 0.011 |
|  |  |  |  |
| Fruiting probability  (Model 5) |  |  |  |
| Intercept | -1.004 | 0.303 | 0.001 |
| *Flw*_t_ | 0.628 | 0.108 | 0.0001 |
| *Grw*_t_ | 0.135 | 0.080 | 0.091 |
| *Grw*_t_ *× Flw*_t_ | -0.096 | 0.033 | 0.003 |
|  |  |  |  |
| Fruiting quantity |  |  |  |
| Intercept | -0.233 | 0.080 | 0.004 |
| *Flw*_t_ | 0.273 | 0.039 | 0.0001 |
| *Grw*_t_ | 0.065 | 0.045 | 0.150 |
| *Grw*_t_ *× Flw*_t_ | -0.029 | 0.012 | 0.017 |
|  |  |  |  |
| Juvenile survival probability  (Model 2) | |  |  |
| Intercept | 2.363 | 0.237 | 0.0001 |
| *Siz*_t_ | -0.808 | 0.209 | 0.0001 |
| *Grw*_t_ | -1.483 | 0.255 | 0.0001 |
| *Siz*_t_ *× Grw*_t_ | 0.836 | 0.168 | 0.0001 |
|  |  |  |  |
| Juvenile sprouting probability  (model 2) | |  |  |
| Intercept | 0.408 | 0.248 | 0.100 |
| *Siz*_t_ | 0.429 | 0.122 | 0.0005 |
|  |  |  |  |
| Juvenile growth (*Siz*_t+1_) | |  |  |
| Intercept | 0.007 | 0.079 | 0.928 |
| *Siz*_t_ | 0.241 | 0.077 | 0.002 |
| *Grw*_t_ | -0.185 | 0.049 | 0.0002 |
| *Siz*_t_ *× Grw*_t_ | 0.098 | 0.042 | 0.018 |
|  |  |  |  |
