# Supplementary material for: Predicting evolution in response to climate change: the example of sprouting probability in three dormancy-prone orchid species
Source: R Soc Open Sci. 2017 Jan 18;4(1):160647. doi: 10.1098/rsos.160647 (PMC5319331; doi:10.1098/rsos.160647)
Supplement: Table S2. Best-fit parameters for C.candidum [file rsos160647supp9.docx]

**Table S2.** Estimates of the effects of factors on demographic parameters in a population of *Cypripedium candidum* monitored for 21 years at Gavin Prairie Nature Preserve, Lake County, Illinois, USA. Fixed factors include size in year *t* (*Siz*_t_), growth between years *t*-1 and *t* (*Grw*_t_, given as *Siz*_t_-*Siz*_t-1_), flowering status in years *t*-1 and *t* (*Flw*_yn,t-1_ and *Flw*_yn,t_), number of flowers in year *t* (*Flw*_t_), total annual precipitation in year *t* and year *t*+1 (*TPCP*_t_ and *TPCP*_t+1_, respectively), and the number of days with temperatures below 0°C in year *t* and year *t*+1 (*DT32*_t_ and *DT32*_t+1_, respectively). Size was measured as the number of aboveground sprouts. Year was included as a random effect in all models. As seedlings could not be tracked longitudinally, no models are presented for that stage. Mixed model analysis was conducted using function *glmer* in package *lme4* in *R* 3.2.2 ([Bates, Maechler & Bolker 2012](#_ENREF_1); [R Core Team 2012](#_ENREF_22)). Estimates are derived from the model with the lowest AICc unless noted, in which case they are derived from an equally parsimonious model with fewer parameters.

| *C. candidum* models | | | |
| --- | --- | --- | --- |
| Effects | Estimate | SE | *P* ≤ |
| Adult survival probability  (Model 3) | |  |  |
| Intercept | 12.084 | 3.404 | 0.0004 |
| *DT32*_t+1_ | -0.068 | 0.024 | 0.004 |
| *Siz*_t_ | 0.534 | 0.137 | 0.0001 |
| *Grw*_t_ | -1.713 | 0.548 | 0.002 |
| *Grw*_t_ *× DT32*_t+1_ | 0.008 | 0.004 | 0.037 |
|  |  |  |  |
| Adult sprouting probability  (Model 4) | |  |  |
| Intercept | 0.410 | 2.052 | 0.842 |
| *DT32*_t+1_ | 0.017 | 0.013 | 0.187 |
| *TPCP*_t+1_ | -0.022 | 0.011 | 0.054 |
| *Siz*_t_ | 0.717 | 0.116 | 0.0001 |
| *Grw*_t_ | -1.508 | 0.414 | 0.0003 |
| *Flw*_yn,t_ | 0.763 | 0.319 | 0.017 |
| *Flw*_yn_,_t-1_ | 0.093 | 0.259 | 0.719 |
| *Flw*_yn,t-1_ *× Grw*_t_ | 0.313 | 0.096 | 0.001 |
| *Siz*_t_ *× Flw*_yn_,_t_ | -0.406 | 0.119 | 0.0007 |
| *Grw*_t_ *× DT32*_t+1_ | 0.008 | 0.003 | 0.006 |
|  |  |  |  |
| Adult growth (*Siz*_t+1_)  (Model 2) |  |  |  |
| Intercept | 1.426 | 0.405 | 0.0004 |
| *DT32*_t+1_ | -0.007 | 0.003 | 0.014 |
| *Siz*_t_ | 0.174 | 0.012 | 0.0001 |
| *Grw*_t_ | -0.253 | 0.065 | 0.0001 |
| *Flw*_yn_,_t_ | -0.788 | 0.415 | 0.058 |
| *Flw*_yn_,_t-1_ | 0.197 | 0.044 | 0.0001 |
| *Grw*_t_ *× DT32*_t+1_ | 0.001 | 0.0005 | 0.002 |
| *Flw*_yn,t_ *× DT32*_t+1_ | 0.008 | 0.003 | 0.008 |
| *Siz*_t_ *× Flw*_yn_,_t_ | -0.064 | 0.012 | 0.0001 |
| *Grw*_t_ *× Flw*_yn_,_t_ | 0.063 | 0.011 | 0.0001 |
| *Grw*_t_ *× Flw*_yn_,_t-1_ | -0.047 | 0.011 | 0.0001 |
|  |  |  |  |
| Flowering probability  (Model 4) |  |  |  |
| Intercept | -1.457 | 0.244 | 0.0001 |
| *Siz*_t_ | 0.130 | 0.031 | 0.0001 |
| *Flw*_yn_,_t_ | 1.313 | 0.183 | 0.0001 |
| *Flw*_yn_,_t-1_ | 0.945 | 0.181 | 0.0001 |
|  |  |  |  |
| Flowering quantity  (Model 3) |  |  |  |
| Intercept | 0.425 | 0.229 | 0.064 |
| *TPCP*_t_ | -0.003 | 0.002 | 0.161 |
| *Siz*_t_ | 0.118 | 0.006 | 0.0001 |
| *Grw*_t_ | 0.053 | 0.019 | 0.004 |
| *Flw*_yn_,_t-1_ | 0.248 | 0.076 | 0.001 |
| *Siz*_t_ *× Grw*_t_ | -0.003 | 0.001 | 0.004 |
|  |  |  |  |
| Fruiting probability |  |  |  |
| Intercept | -1.361 | 0.432 | 0.002 |
| *Flw*_t_ | 0.250 | 0.063 | 0.0001 |
|  |  |  |  |
| Fruiting quantity |  |  |  |
| Intercept | 0.152 | 0.118 | 0.198 |
| *Flw*_t_ | 0.101 | 0.013 | 0.0001 |
|  |  |  |  |
| Juvenile survival probability  (Model 2) | |  |  |
| Intercept | 7.582 | 4.368 | 0.083 |
| *TPCP*_t+1_ | -0.072 | 0.047 | 0.121 |
| *Siz*_t_ | 2.958 | 1.636 | 0.071 |
| *Grw*_t_ | -2.251 | 1.191 | 0.059 |
|  |  |  |  |
| Juvenile sprouting probability  (Model 2) | |  |  |
| Intercept | 9.788 | 6.611 | 0.139 |
| *DT32*_t+1_ | -0.065 | 0.048 | 0.179 |
| *Siz*_t_ | -18.621 | 7.636 | 0.015 |
| *Siz*_t_ *× DT32*_t+1_ | 0.141 | 0.057 | 0.013 |
|  |  |  |  |
| Juvenile growth (*Siz*_t+1_)  (Model 2) | |  |  |
| Intercept | 0.554 | 0.097 | 0.0001 |
| *Grw*_t_ | -0.125 | 0.012 | 0.0001 |
|  |  |  |  |
