# Supplementary material for: Predicting evolution in response to climate change: the example of sprouting probability in three dormancy-prone orchid species
Source: R Soc Open Sci. 2017 Jan 18;4(1):160647. doi: 10.1098/rsos.160647 (PMC5319331; doi:10.1098/rsos.160647)
Supplement: Table S3. Best-fit parameters for O. sphegodes [file rsos160647supp10.docx]

**Table S3.** Estimates of the effects of factors on demographic parameters in a population of *Ophrys sphegodes*, monitored for 32 years at Castle Hill National Nature Reserve in Sussex, England. Fixed factors include size in year *t* (*Siz*_t_), growth between years *t*-1 and *t* (*Grw*_t_, given as *Siz*_t_-*Siz*_t-1_), flowering status in year *t* (*Flw*_yn,t_), number of flowers in year *t* (*Flw*_t_), the number of years since the first observation of the individual (*TSE*_t_, abbreviated from *Time Since Entry*, which is a proxy for age), total precipitation from the start of February until the end of May of year *t* and *t*+1 (*SpPrec*_t_ and *SpPrec*_t+1_, respectively), the number of hours of sunshine in April and May of year *t* and *t*+1 (*Sun*_t_ and *Sun*_t+1_, respectively), as well as all possible interactions in the given model. Size was measured as the number of leaves. Year was included as a random effect in all models. As seedlings could not be tracked longitudinally, no models are presented for that stage. Mixed model analysis was conducted using function *glmer* in package *lme4* in *R* 3.2.2 ([Bates, Maechler & Bolker 2012](#_ENREF_1); [R Core Team 2012](#_ENREF_22)). Estimates are derived from the model with the lowest AICc unless noted, in which case they are derived from an equally parsimonious model with fewer parameters.

| *O. sphegodes* models | | | |
| --- | --- | --- | --- |
| Effects | Estimate | SE | *P* ≤ |
| Survival probability |  |  |  |
| Intercept | 0.867 | 1.024 | 0.397 |
| *SpPrec* _t+1_ | 0.002 | 0.002 | 0.313 |
| *Sun*_t+1_ | 0.001 | 0.002 | 0.454 |
| *Siz*_t_ | 0.195 | 0.150 | 0.191 |
| *TSE*_t_ | -0.007 | 0.074 | 0.921 |
| *Flw*_yn_,_t_ | -1.197 | 0.225 | 0.0001 |
| *Siz*_t_ *× Flw*_yn_,_t_ | 0.526 | 0.041 | 0.0001 |
| *Flw*_yn_,_t_ *× SpPrec*_t+1_ | -0.003 | 0.001 | 0.002 |
| *Flw*_yn_,_t_ *× TSE*_t_ | -0.052 | 0.015 | 0.0004 |
| *Siz*_t_ *× Sun*_t+1_ | -0.001 | 0.0003 | 0.0003 |
| *Siz*_t_ *× TSE*_t_ | -0.037 | 0.006 | 0.0001 |
| *Sun*_t+1_ *× TSE*_t_ | 0.0006 | 0.0002 | 0.0006 |
|  |  |  |  |
| Sprouting probability  (Model 6) |  |  |  |
| Intercept | 0.237 | 0.200 | 0.238 |
| *Siz*_t_ | 0.169 | 0.014 | 0.0001 |
|  |  |  |  |
| Growth (*Siz*_t+1_) |  |  |  |
| Intercept | 0.940 | 0.110 | 0.0001 |
| *SpPrec* _t+1_ | 0.001 | 0.001 | 0.030 |
| *Siz*_t_ | 0.016 | 0.004 | 0.0002 |
|  |  |  |  |
| Flowering probability  (Model 3) |  |  |  |
| Intercept | 2.771 | 2.172 | 0.202 |
| *Sun*_t+1_ | -0.006 | 0.005 | 0.175 |
| *Siz*_t_ | 0.291 | 0.078 | 0.005 |
| *Flw*_yn_,_t_ | 1.558 | 0.602 | 0.010 |
| *Flw*_yn_,_t_ *× Sun*_t+1_ | -0.003 | 0.001 | 0.031 |
| *Siz*_t_ *× SpPrec* _t+1_ | -0.0007 | 0.0004 | 0.049 |
| *Sun*_t+1_ *× SpPrec*_t+1_ | 0.000003 | 0.00001 | 0.789 |
|  |  |  |  |
| Flowering quantity |  |  |  |
| Intercept | 0.676 | 0.040 | 0.0001 |
| *Siz*_t_ | 0.073 | 0.010 | 0.0001 |
|  |  |  |  |
