# Supplementary material for: Predicting evolution in response to climate change: the example of sprouting probability in three dormancy-prone orchid species
Source: R Soc Open Sci. 2017 Jan 18;4(1):160647. doi: 10.1098/rsos.160647 (PMC5319331; doi:10.1098/rsos.160647)
Supplement: Table S4. Model selection table for C. parviforum [file rsos160647supp11.docx]

**Table S4.** Results of model selection in linear modeling of vital rates in *Cypripedium parviflorum*, monitored for 21 years at Gavin Prairie Nature Preserve, Lake County, Illinois, USA. Top ten general linear mixed models (GLMMs) are presented for each demographic parameter. Parameters included in each model are marked with a +. Fixed factors include size in year *t* (*Siz*_t_), growth between years *t*-1 and *t* (*Grw*_t_, given as *Siz*_t_-*Siz*_t-1_), flowering status in years *t*-1 and *t* (*Flw*_yn,t-1_ and *Flw*_yn,t_), number of flowers in year *t* (*Flw*_t_), total annual precipitation in year *t* and year *t*+1 (*TPCP*_t_ and *TPCP*_t+1_, respectively), and the number of days with temperatures below 0°C in year *t* and year *t*+1 (*DT32*_t_ and *DT32*_t+1_, respectively). Size was measured as the number of aboveground sprouts. Year was included as a random effect in all models.

| Effects | Model #1  (best-fit) | #2 | #3 | #4 | #5 | #6 | #7 | #8 | #9 | #10 |
| --- | --- | --- | --- | --- | --- | --- | --- | --- | --- | --- |
| Adult survival probability | |  |  |  |  |  |  |  |  |  |
| *DT32*_t+1_ | + | + | + | + | + | + | + | + | + | + |
| *TPCP*_t+1_ | + | + | + | + | + | + | + | + | + | + |
| *Siz*_t_ | + | + | + | + | + | + | + | + | + | + |
| *Grw*_t_ | + | + | + | + | + | + | + | + | + | + |
| *Flw*_yn_,_t_ | + | + | + | + | + | + | + | + | + | + |
| *Flw*_yn_,_t-1_ | + | + | + | + | + | + | + | + | + | + |
| *Siz*_t_ *× TPCP*_t+1_ | + | + | + | + | + | + | + | + | + | + |
| *Siz*_t_ *× DT32*_t+1_ |  |  |  | + |  |  |  |  | + |  |
| *Siz*_t_ *× Grw*_t_ | + | + | + | + | + | + | + | + | + | + |
| *Siz*_t_ *× Flw*_yn_,_t_ | + | + | + | + | + | + | + | + | + | + |
| *Flw*_yn_,_t-1_ *× Flw*_yn_,_t_ |  |  |  |  |  |  |  | + |  |  |
| *Flw*_yn_,_t_ *× DT32*_t+1_ |  |  |  |  |  |  |  |  |  | + |
| *Flw*_yn_,_t_ *× TPCP*_t+1_ |  |  | + |  | + |  |  |  |  |  |
| *Grw*_t_ *× DT32*_t+1_ | + | + | + | + | + | + | + | + | + | + |
| *Grw*_t_ *× TPCP*_t+1_ |  |  |  |  |  |  |  |  |  |  |
| *Grw*_t_ *× Flw*_yn_,_t_ |  | + | + |  |  |  | + | + | + |  |
| *Grw*_t_ *× Flw*_yn_,_t-1_ | + | + | + | + | + | + | + | + | + | + |
| *DT32*_t+1_ *× TPCP*_t+1_ |  |  |  |  |  | + | + |  |  |  |
| Df | 13 | 14 | 15 | 14 | 14 | 14 | 15 | 15 | 15 | 14 |
| ΔAICc | 0 | 0.02 | 1.16 | 1.24 | 1.24 | 1.26 | 1.29 | 1.32 | 1.38 | 1.69 |
|  |  |  |  |  |  |  |  |  |  |  |
| Adult sprouting probability | |  |  |  |  |  |  |  |  |  |
| *DT32*_t+1_ | + | + | + | + | + | + | + | + | + | + |
| *TPCP*_t+1_ | + | + | + | + | + | + | + | + | + | + |
| *Siz*_t_ | + | + | + | + | + | + | + | + | + | + |
| *Grw*_t_ | + | + | + | + | + | + | + | + | + | + |
| *Flw*_yn_,_t_ | + | + | + | + | + | + | + | + | + | + |
| *Flw*_yn_,_t-1_ |  | + | + |  | + | + | + |  | + | + |
| *Siz*_t_ *× DT32*_t+1_ |  |  |  | + | + |  | + |  |  | + |
| *Siz*_t_ *× Grw*_t_ | + | + |  | + | + | + |  | + | + | + |
| *Siz*_t_ *× Flw*_yn_,_t_ | + | + | + | + | + |  | + | + | + |  |
| *Flw*_yn_,_t-1_ *× Flw*_yn_,_t_ |  | + | + |  | + | + | + |  | + | + |
| *Grw*_t_ *× DT32*_t+1_ | + | + | + | + | + | + | + | + | + | + |
| *Grw*_t_ *× TPCP*_t+1_ | + | + | + | + | + | + | + | + | + | + |
| *Grw*_t_ *× Flw*_yn_,_t_ |  | + | + |  | + | + | + | + |  | + |
| Df | 11 | 14 | 13 | 12 | 15 | 13 | 14 | 12 | 13 | 14 |
| ΔAICc | 0 | 0.39 | 0.62 | 0.68 | 1.08 | 1.10 | 1.27 | 1.45 | 1.60 | 1.66 |
|  |  |  |  |  |  |  |  |  |  |  |
| Adult growth (*Siz*_t+1_) |  |  |  |  |  |  |  |  |  |  |
| *DT32*_t+1_ |  | + |  | + | + | + | + |  | + | + |
| *TPCP*_t+1_ | + | + | + | + | + | + | + | + | + | + |
| *Siz*_t_ | + | + | + | + | + | + | + | + | + | + |
| *Grw*_t_ | + | + | + | + | + | + | + | + | + | + |
| *Flw*_yn_,_t_ | + | + | + | + | + | + | + | + | + | + |
| *Flw*_yn_,_t-1_ | + | + | + | + | + | + | + | + | + | + |
| *Siz*_t_ *× TPCP*_t+1_ | + | + | + | + | + | + | + | + | + | + |
| *Siz*_t_ *× DT32*_t+1_ |  |  |  | + |  | + |  |  |  |  |
| *Siz*_t_ *× Grw*_t_ | + | + | + | + | + | + | + | + | + | + |
| *Siz*_t_ *× Flw*_yn_,_t_ | + | + | + | + | + | + | + | + | + | + |
| *Flw*_yn_,_t-1_ *× Flw*_yn_,_t_ |  |  | + |  | + | + |  | + |  | + |
| *Flw*_yn_,_t_ *× DT32*_t+1_ |  |  |  |  |  |  | + |  |  |  |
| *Flw*_yn_,_t_ *× TPCP*_t+1_ | + | + | + | + | + | + | + |  | + | + |
| *Grw*_t_ *× DT32*_t+1_ |  | + |  | + | + | + | + |  |  |  |
| *Grw*_t_ *× Flw*_yn_,_t_ | + | + | + | + | + | + | + | + | + | + |
| *Grw*_t_ *× Flw*_yn_,_t-1_ | + | + | + | + | + | + | + | + | + | + |
| Df | 13 | 15 | 14 | 16 | 16 | 17 | 16 | 13 | 14 | 15 |
| ΔAICc | 0 | 0.06 | 0.10 | 0.29 | 0.47 | 0.83 | 1.60 | 1.68 | 1.69 | 1.78 |
|  |  |  |  |  |  |  |  |  |  |  |
| Flowering probability |  |  |  |  |  |  |  |  |  |  |
| *DT32*_t+1_ |  | + | + |  | + | + |  | + |  | + |
| *Siz*_t_ | + | + | + | + | + | + | + | + | + | + |
| *Grw*_t_ | + | + | + | + | + | + | + | + | + | + |
| *Flw*_yn_,_t_ | + | + | + | + | + | + | + | + | + | + |
| *Flw*_yn_,_t-1_ | + | + | + | + | + | + | + | + | + | + |
| *Siz*_t_ *× DT32*_t+1_ |  | + | + |  | + | + |  | + |  | + |
| *Siz*_t_ *× Grw*_t_ | + | + | + | + | + | + | + | + | + | + |
| *Siz*_t_ *× Flw*_yn_,_t_ |  |  |  | + | + | + |  |  | + |  |
| *Flw*_yn_,_t-1_ *× Flw*_yn_,_t_ |  |  |  |  |  |  | + | + |  | + |
| *Grw*_t_ *× DT32*_t+1_ |  | + |  |  | + |  |  | + |  |  |
| *Grw*_t_ *× Flw*_yn_,_t_ |  |  |  |  |  |  |  |  | + |  |
| *Grw*_t_ *× Flw*_yn_,_t-1_ | + | + | + | + | + | + | + | + | + | + |
| Df | 8 | 11 | 10 | 9 | 12 | 11 | 9 | 12 | 10 | 11 |
| ΔAICc | 0 | 0.22 | 0.41 | 0.56 | 0.78 | 1.07 | 1.23 | 1.44 | 1.53 | 1.62 |
|  |  |  |  |  |  |  |  |  |  |  |
| Flowering quantity |  |  |  |  |  |  |  |  |  |  |
| *DT32*_t_ |  | + |  | + | + | + | + |  | + |  |
| *TPCP*_t_ |  |  | + |  | + |  |  | + | + | + |
| *Siz*_t_ | + | + | + | + | + | + | + | + | + | + |
| *Grw*_t_ | + | + | + | + | + | + | + | + | + | + |
| *Flw*_yn_,_t-1_ | + | + | + | + | + | + | + | + | + | + |
| *Siz*_t_ *× DT32*_t_ |  |  |  |  |  |  | + |  |  |  |
| *Siz*_t_ *× Grw*_t_ | + | + | + | + | + | + | + | + | + | + |
| *Flw*_yn_,_t-1_ *× Flw*_yn_,_t_ |  |  |  |  |  |  |  |  |  |  |
| *Flw*_yn_,_t-1_ *× DT32*_t_ |  |  |  |  |  | + |  |  |  |  |
| *Flw*_yn_,_t-1_ *× TPCP*_t_ |  |  |  |  |  |  |  | + | + |  |
| *Grw*_t_ *× DT32*_t_ |  |  |  | + |  |  |  |  |  |  |
| *Grw*_t_ *× TPCP*_t_ |  |  |  |  |  |  |  |  |  | + |
| *Grw*_t_ *× Flw*_yn_,_t-1_ | + | + | + | + | + | + | + | + | + | + |
| Df | 7 | 8 | 8 | 9 | 9 | 9 | 9 | 9 | 10 | 9 |
| ΔAICc | 0 | 0.39 | 1.71 | 1.91 | 1.95 | 2.13 | 2.16 | 3.05 | 3.18 | 3.20 |
|  |  |  |  |  |  |  |  |  |  |  |
| Fruiting probability |  |  |  |  |  |  |  |  |  |  |
| *DT32*_t_ | + | + | + |  |  | + |  | + | + | + |
| *TPCP*_t_ |  |  |  |  |  |  |  |  | + |  |
| *Flw*_t_ | + | + | + | + | + | + | + | + | + | + |
| *Grw*_t_ | + | + | + | + | + | + | + | + | + | + |
| *Flw*_yn_,_t-1_ | + |  | + | + |  | + | + |  | + | + |
| *Flw*_t_ *× DT32*_t_ |  |  |  |  |  | + |  | + |  | + |
| *Flw*_t_ *× Grw*_t_ | + | + | + | + | + | + | + | + | + | + |
| *Grw*_t_ *× Flw*_yn_,_t-1_ | + |  |  | + |  | + |  |  | + |  |
| *DT32*_t_ *× TPCP*_t_ |  |  |  |  |  |  |  |  |  |  |
| Df | 8 | 6 | 7 | 7 | 5 | 9 | 6 | 7 | 9 | 8 |
| ΔAICc | 0 | 0.19 | 0.29 | 0.88 | 1.06 | 1.19 | 1.32 | 1.52 | 1.72 | 1.72 |
|  |  |  |  |  |  |  |  |  |  |  |
| Fruiting quantity |  |  |  |  |  |  |  |  |  |  |
| *DT32*_t_ |  | + |  | + |  | + |  | + | + | + |
| *TPCP*_t_ |  |  |  |  | + |  |  |  | + |  |
| *Flw*_t_ | + | + | + | + | + | + | + | + | + | + |
| *Grw*_t_ | + | + | + | + | + | + |  | + | + | + |
| *Flw*_yn_,_t-1_ |  |  | + |  |  | + |  |  |  | + |
| *Flw*_t_ *× DT32*_t_ |  |  |  | + |  |  |  |  |  |  |
| *Flw*_t_ *× Grw*_t_ | + | + | + | + | + | + |  | + | + | + |
| *Flw*_yn_,_t-1_ *× DT32*_t_ |  |  |  |  |  |  |  |  |  | + |
| *Grw*_t_ *× DT32*_t_ |  |  |  |  |  |  |  | + |  |  |
| Df | 5 | 6 | 6 | 7 | 6 | 7 | 3 | 7 | 7 | 8 |
| ΔAICc | 0 | 0.85 | 1.61 | 1.79 | 1.94 | 2.24 | 2.57 | 2.67 | 2.85 | 3.19 |
|  |  |  |  |  |  |  |  |  |  |  |
| Juvenile survival probability | |  |  |  |  |  |  |  |  |  |
| *DT32*_t+1_ | + |  | + | + | + | + |  | + | + |  |
| *TPCP*_t+1_ |  |  |  |  | + | + | + | + | + | + |
| *Siz*_t_ | + | + | + | + | + | + | + | + | + | + |
| *Grw*_t_ | + | + | + | + | + | + | + | + | + | + |
| *Siz*_t_ *× TPCP*_t+1_ |  |  |  |  |  |  |  |  | + |  |
| *Siz*_t_ *× DT32*_t+1_ |  |  | + |  |  |  |  |  |  |  |
| *Siz*_t_ *× Grw*_t_ | + | + | + | + | + | + | + | + | + | + |
| *Grw*_t_ *× DT32*_t+1_ |  |  |  | + |  |  |  |  |  |  |
| *Grw*_t_ *× TPCP*_t+1_ |  |  |  |  |  | + |  |  | + | + |
| *DT32*_t+1_ *× TPCP*_t+1_ |  |  |  |  |  |  |  | + |  |  |
| Df | 6 | 5 | 7 | 7 | 7 | 8 | 6 | 8 | 9 | 7 |
| ΔAICc | 0 | 1.05 | 1.88 | 1.88 | 1.90 | 2.40 | 2.97 | 3.03 | 3.34 | 3.69 |
|  |  |  |  |  |  |  |  |  |  |  |
| Juvenile sprouting probability | |  |  |  |  |  |  |  |  |  |
| *DT32*_t+1_ | + |  | + | + | + |  |  | + | + | + |
| *TPCP*_t+1_ |  |  | + |  |  | + |  | + | + | + |
| *Siz*_t_ | + | + | + | + | + | + | + | + | + | + |
| *Grw*_t_ |  |  |  |  | + |  | + |  |  | + |
| *Siz*_t_ *× TPCP*_t+1_ |  |  |  |  |  |  |  |  |  |  |
| *Siz*_t_ *× DT32*_t+1_ |  |  |  | + |  |  |  |  | + |  |
| *DT32*_t+1_ *× TPCP*_t+1_ |  |  |  |  |  |  |  | + |  |  |
| Df | 4 | 3 | 5 | 5 | 5 | 4 | 4 | 6 | 6 | 6 |
| ΔAICc | 0 | 0.60 | 1.29 | 1.72 | 1.90 | 2.13 | 2.44 | 2.85 | 3.00 | 3.22 |
|  |  |  |  |  |  |  |  |  |  |  |
| Juvenile growth (*Siz*_t+1_) | |  |  |  |  |  |  |  |  |  |
| *DT32*_t+1_ |  | + |  |  | + |  | + | + |  | + |
| *TPCP*_t+1_ |  |  | + | + | + |  |  |  | + | + |
| *Siz*_t_ | + | + | + | + | + | + | + | + | + | + |
| *Grw*_t_ | + | + | + | + | + | + | + | + | + | + |
| *Siz*_t_ *× TPCP*_t+1_ |  |  |  | + |  |  |  |  |  | + |
| *Siz*_t_ *× DT32*_t+1_ |  |  |  |  |  |  |  | + |  |  |
| *Siz*_t_ *× Grw*_t_ | + | + | + | + | + |  | + | + | + | + |
| *Grw*_t_ *× DT32*_t+1_ |  |  |  |  |  |  | + |  |  |  |
| *Grw*_t_ *× TPCP*_t+1_ |  |  |  |  |  |  |  |  | + |  |
| Df | 5 | 6 | 6 | 7 | 7 | 4 | 7 | 7 | 7 | 8 |
| ΔAICc | 0 | 1.51 | 1.57 | 2.97 | 3.05 | 3.33 | 3.36 | 3.52 | 3.60 | 4.52 |
|  |  |  |  |  |  |  |  |  |  |  |
