# Supplementary material for: Predicting evolution in response to climate change: the example of sprouting probability in three dormancy-prone orchid species
Source: R Soc Open Sci. 2017 Jan 18;4(1):160647. doi: 10.1098/rsos.160647 (PMC5319331; doi:10.1098/rsos.160647)
Supplement: Table S6. Model selection table for O. sphegodes [file rsos160647supp13.docx]

**Table S6.** Results of model selection in linear modeling of vital rates in *Ophrys sphegodes*, monitored for 32 years at Castle Hill National Nature Reserve in Sussex, England. Top ten general linear mixed models (GLMMs) are presented for each demographic parameter. Parameters included in each model are marked with a +. Fixed factors include size in year *t* (*Siz*_t_), growth between years *t*-1 and *t* (*Grw*_t_, given as *Siz*_t_-*Siz*_t-1_), flowering status in year *t* (*Flw*_yn,t_), number of flowers in year *t* (*Flw*_t_), the number of years since the first observation of the individual (*TSE*_t_, abbreviated from *Time Since Entry*, which is a proxy for age), total precipitation from the start of February until the end of May of year *t* and *t*+1 (*SpPrec*_t_ and *SpPrec*_t+1_, respectively), the number of hours of sunshine in April and May of year *t* and *t*+1 (*Sun*_t_ and *Sun*_t+1_, respectively), as well as all possible interactions in the given model. Size was measured as the number of leaves. Year was included as a random effect in all models.

| Effects | Model #1  (best-fit) | #2 | #3 | #4 | #5 | #6 | #7 | #8 | #9 | #10 |
| --- | --- | --- | --- | --- | --- | --- | --- | --- | --- | --- |
| Adult survival probability | |  |  |  |  |  |  |  |  |  |
| *Sun*_t+1_ | + | + | + | + | + | + | + | + | + | + |
| *SpPrec*_t+1_ | + | + | + | + | + | + | + | + | + | + |
| *Siz*_t_ | + | + | + | + | + | + | + | + | + | + |
| *TSE*_t_ | + | + | + | + | + | + | + | + | + | + |
| *Flw*_yn_,_t_ | + | + | + | + | + | + | + | + | + | + |
| *Siz*_t_ *× SpPrec*_t+1_ |  | + |  |  |  | + | + | + |  |  |
| *Siz*_t_ *× Sun*_t+1_ | + | + | + | + | + | + | + | + | + | + |
| *Siz*_t_ *× TSE*_t_ | + | + | + | + | + | + | + | + | + | + |
| *Siz*_t_ *× Flw*_yn_,_t_ | + | + | + | + | + | + | + | + | + | + |
| *Flw*_yn_,_t_ *× Sun*_t+1_ |  |  |  | + |  |  |  | + | + |  |
| *Flw*_yn_,_t_ *× SpPrec*_t+1_ | + | + | + | + | + | + | + | + | + | + |
| *TSE*_t_ *× Sun*_t+1_ | + | + | + | + | + | + | + | + | + | + |
| *TSE*_t_ *× SpPrec*_t+1_ |  |  | + |  |  | + |  |  | + | + |
| *TSE*_t_ *× Flw*_yn_,_t_ | + | + | + | + | + | + | + | + | + | + |
| *Sun*_t+1_ *× SpPrec*_t+1_ |  |  |  |  | + |  | + |  |  | + |
| Df | 13 | 14 | 14 | 14 | 14 | 15 | 15 | 15 | 15 | 15 |
| ΔAICc | 0 | 1.00 | 1.55 | 1.91 | 1.93 | 2.54 | 2.94 | 2.96 | 3.47 | 3.48 |
|  |  |  |  |  |  |  |  |  |  |  |
| Adult sprouting probability | |  |  |  |  |  |  |  |  |  |
| *Sun*_t+1_ | + | + |  | + | + |  | + | + | + | + |
| *Siz*_t_ | + | + | + | + | + | + | + | + | + | + |
| *TSE*_t_ | + | + | + | + | + |  |  | + | + |  |
| *Flw*_yn_,_t_ | + | + | + | + | + |  |  | + | + | + |
| *Siz*_t_ *× Sun*_t+1_ | + |  |  | + |  |  |  |  | + | + |
| *Siz*_t_ *× TSE*_t_ |  |  |  |  |  |  |  |  | + |  |
| *Flw*_yn_,_t_ *× Sun*_t+1_ | + | + |  | + |  |  |  | + | + | + |
| *TSE*_t_ *× Sun*_t+1_ |  |  |  | + |  |  |  | + |  |  |
| *TSE*_t_ *× Flw*_yn_,_t_ | + | + | + | + | + |  |  | + | + |  |
| Df | 9 | 8 | 6 | 10 | 7 | 3 | 4 | 9 | 10 | 7 |
| ΔAICc | 0 | 0.96 | 1.07 | 1.10 | 1.24 | 1.28 | 1.42 | 1.55 | 1.60 | 1.81 |
|  |  |  |  |  |  |  |  |  |  |  |
| Adult growth (*Siz*_t+1_) |  |  |  |  |  |  |  |  |  |  |
| *Sun*_t+1_ |  | + |  | + |  |  | + |  |  | + |
| *SpPrec*_t+1_ | + | + | + |  | + | + | + |  | + | + |
| *Siz*_t_ | + | + | + | + | + | + | + | + | + | + |
| *TSE*_t_ |  |  |  |  |  | + |  |  | + |  |
| *Flw*_yn_,_t_ |  |  |  |  | + |  |  |  |  |  |
| *Siz*_t_ *× SpPrec*_t+1_ |  |  | + |  |  |  | + |  |  |  |
| *Siz*_t_ *× TSE*_t_ |  |  |  |  |  |  |  |  | + |  |
| *Sun*_t+1_ *× SpPrec*_t+1_ |  |  |  |  |  |  |  |  |  | + |
| Df | 4 | 5 | 5 | 4 | 5 | 5 | 6 | 3 | 6 | 6 |
| ΔAICc | 0 | 1.00 | 1.02 | 1.39 | 1.94 | 2.00 | 2.06 | 2.44 | 2.66 | 2.83 |
|  |  |  |  |  |  |  |  |  |  |  |
| Flowering probability |  |  |  |  |  |  |  |  |  |  |
| *Sun*_t+1_ | + | + | + | + | + | + | + | + | + | + |
| *SpPrec*_t+1_ | + | + | + | + | + | + | + | + | + | + |
| *Siz*_t_ | + | + | + | + | + | + | + | + | + | + |
| *TSE*_t_ | + | + |  | + | + |  | + | + | + | + |
| *Flw*_yn_,_t_ | + | + | + | + | + | + | + | + | + | + |
| *Siz*_t_ *× SpPrec*_t+1_ | + | + | + | + | + | + | + |  | + |  |
| *Siz*_t_ *× Flw*_yn_,_t_ |  |  |  | + | + | + |  |  |  |  |
| *Flw*_yn_,_t_ *× Sun*_t+1_ | + | + | + | + | + | + | + | + | + | + |
| *Flw*_yn_,_t_ *× SpPrec*_t+1_ |  |  |  |  |  |  |  |  | + |  |
| *TSE*_t_ *× Sun*_t+1_ |  | + |  |  | + |  |  | + |  |  |
| *TSE*_t_ *× SpPrec*_t+1_ |  |  |  |  |  |  | + |  |  |  |
| *Sun*_t+1_ *× SpPrec*_t+1_ | + | + | + | + | + | + | + | + | + | + |
| Df | 10 | 11 | 9 | 11 | 12 | 10 | 11 | 10 | 11 | 9 |
| ΔAICc | 0 | 0.18 | 0.34 | 1.41 | 1.54 | 1.62 | 1.79 | 1.80 | 1.80 | 1.82 |
|  |  |  |  |  |  |  |  |  |  |  |
| Flowering quantity |  |  |  |  |  |  |  |  |  |  |
| *Sun*_t_ |  |  |  | + | + |  |  | + |  | + |
| *SpPrec*_t_ |  |  | + |  |  |  | + |  | + | + |
| *Siz*_t_ | + | + | + | + | + | + | + | + | + | + |
| *TSE*_t_ |  | + |  |  |  | + | + | + |  |  |
| *Siz*_t_ *× SpPrec*_t_ |  |  |  |  |  |  |  |  | + |  |
| *Siz*_t_ *× Sun*_t_ |  |  |  |  | + |  |  |  |  |  |
| *Siz*_t_ *× TSE*_t_ |  |  |  |  |  | + |  |  |  |  |
| Df | 3 | 4 | 4 | 4 | 5 | 5 | 5 | 5 | 5 | 5 |
| ΔAICc | 0 | 1.67 | 1.94 | 1.99 | 3.45 | 3.47 | 3.60 | 3.67 | 3.93 | 3.94 |
|  |  |  |  |  |  |  |  |  |  |  |
